# Indirect two-sided relative ranking: a robust similarity measure for gene expression data

**DOI:** 10.1186/1471-2105-11-137

**Published:** 2010-03-17

**Authors:** Louis Licamele, Lise Getoor

**Affiliations:** 1 Computer Science Department, University of Maryland, College Park, USA; 2 Informatics Department, Vanda Pharmaceuticals, Inc., Rockville, USA

## Abstract

**Background:**

There is a large amount of gene expression data that exists in the public domain. This data has been generated under a variety of experimental conditions. Unfortunately, these experimental variations have generally prevented researchers from accurately comparing and combining this wealth of data, which still hides many novel insights.

**Results:**

In this paper we present a new method, which we refer to as indirect two-sided relative ranking, for comparing gene expression profiles that is robust to variations in experimental conditions. This method extends the current best approach, which is based on comparing the correlations of the up and down regulated genes, by introducing a comparison based on the correlations in rankings across the entire database. Because our method is robust to experimental variations, it allows a greater variety of gene expression data to be combined, which, as we show, leads to richer scientific discoveries.

**Conclusions:**

We demonstrate the benefit of our proposed indirect method on several datasets. We first evaluate the ability of the indirect method to retrieve compounds with similar therapeutic effects across known experimental barriers, namely vehicle and batch effects, on two independent datasets (one private and one public). We show that our indirect method is able to significantly improve upon the previous state-of-the-art method with a substantial improvement in recall at rank 10 of 97.03% and 49.44%, on each dataset, respectively. Next, we demonstrate that our indirect method results in improved accuracy for classification in several additional datasets. These datasets demonstrate the use of our indirect method for classifying cancer subtypes, predicting drug sensitivity/resistance, and classifying (related) cell types. Even in the absence of a known (i.e., labeled) experimental barrier, the improvement of the indirect method in each of these datasets is statistically significant.

## Background

There is a large amount of gene expression data, generated from microarray experiments, that exists in the public domain. Gene expression microarrays attempt to measure the amount of mRNA that is transcribed. This gives an estimate of the amount of protein that will be translated from this mRNA. Proteins are responsible for most of the work that is done in the cell, whether it is breaking down compounds, signaling other cells or pathways, or even making up the infrastructure and machinery to continue to transcribe DNA into mRNA. Traditionally, gene expression profiling has been used to understand the underlying mechanism of biological processes and pathways [1,2], to segment and explain diseases and their subtypes [3,4], and to predict cancer prognosis [5,6]. In addition, because it represents how the cell responds to each compound, gene expression data may be a good source for investigating whether two drugs could have a similar therapeutic effect [7].

Unfortunately, gene expression data is inherently complex and difficult to analyze and compare. First, there are many factors that complicate the process including post-transcriptional modification (e.g., splicing), degradation of the mRNA, changes in the translation rates from mRNA to polypeptide chains, as well as post-translational modification (e.g., phosphorylation). Second, the existing data has been generated by many different laboratories across the world in a variety of experiments. These experiments can be testing many different hypotheses, such as the effect of a drug, i.e., which pathways and genes are affected by the drug, or the cause of a disease, i.e., which pathways and genes are differentiated in affected individuals. Different experimental conditions are likely to result in confounding effects on the gene expression profiles.

Historically, when researchers compare gene expression profiles, they limit themselves to data generated under similar experimental conditions. Recently, researchers at the Broad Institute developed a new approach for detecting gene expression similarity. Their tool, the Connectivity-Map (CMAP) [8], addresses the problem of comparing gene expression profiles generated under diverse experimental conditions. Similar to Natsoulis et al. [7], the CMAP approach relies on both positive (reward) and negative (penalty) genes/probes. This builds on Golub et al. [3], who demonstrated using a weighted voting scheme to select gene lists in the context of classifying cancer. Unlike these previous methods, Lamb et al. [8] use a distribution statistic to compare the ranked lists of expression probes. They show that this method is able to overcome some of the experimental noise that can affect the gene expression profile. This noise can be from a wide range of confounding factors such as the vehicle used to deliver the compound or the cell line used for the experiment.

In this paper, we introduce a new similarity measure for comparing ranked lists. Unlike the CMAP approach, which performs a direct comparison of the gene expression profiles, our approach captures the correlations in rankings between the target pair and the rest of the database. This results in a method which is more robust and is able to better cope with experimental barriers such as vehicle and batch effects. We evaluate the ability of this method for both retrieval and classification in several datasets. Our contributions include:

• We formalize the problem of determining similarity in gene expression data as a comparison of ranked lists.

• We describe the CMAP approach, a direct approach, to comparing ranked lists.

• We introduce our novel indirect approach to comparing ranked lists.

• We evaluate both methods on a two drug discovery datasets, and show how our new method is able to overcome experimental noise and obtain 97.03% and 49.44% improvement in recall of similar drugs over the direct approach.

• We further evaluate our method on three additional datasets and demonstrate the ability of our indirect method to work in other gene expression tasks, e.g., classifying cancer subtypes, predicting drug sensitivity/resistance, and lastly classifying (related) cell types.

## Results

### Problem Definition

Given a database *D *of treatments, i.e., drugs or other compounds, *D *= *t*_1_, ..., *t*_*n*_, we are interest in querying the database with a selected query treatment and returning other similar treatments. Typically we know the therapeutic use or indication for the query, but may not have complete therapeutic information for all the entries in the database. We are trying to discover other drugs or treatments, perhaps originally developed for a different therapeutic purpose, that are likely to also share the same therapeutic properties as the query. These drugs then are good candidates for further evaluation of a new use.

More specifically, for each treatment instance *t *in the database, there is both general information about the experimental conditions of the sample, as well as the actual experiment data from the microarray itself. The microarray data consists of a collection of probe sets, *probes*(*t*). Each probe *p *∈ *probes*(*t*) measures the match to a particular genomic sequence. For each probe *p*, there is a raw expression value *EV *(*p*) (calculated using MAS 5 algorithm [9]), as well as an amplitude *A*(*p*) (the difference compared to control). The control is a reference baseline which is the average expression value calculated from multiple untreated samples run within the same vehicle and batch. Information specific to the treatment, i.e., the name of the drug, the therapeutic class (class) and subclass (subclass) as defined by the Physicians Desktop Reference (PDR) is also represented. Additionally, there is information that describes the experimental conditions of the sample, specifically the molar amount of substance (mol), the vehicle used for delivery of the drug (e.g., water, EtOH, MeOH, DMSO) and the batch or round in which the sample was run.

We are interested in retrieving treatments *t *that are similar in some way to a query treatment *q*. We measure similarity based on the probes of *t *and *q*. Rather than measuring the absolute similarity in expression levels, we compare the *ranking *of the probes. Using the ranks allows for a nonparametric comparison of the gene expression profiles. Nonparametric methods have been shown to work well for detecting differentially expressed genes in microarray data [10-12]. As mentioned above, probes have both a raw expression value and an amplitude. This ranking can be done based on either the raw value or the amplitude. We utilize the amplitude because it measures the treatment effect. We use *r*(*p, probes*(*t*)) to denote the rank of *p *in *probes*(*t*); i.e., if the probes are sorted in order of their amplitude, then the rank is the position of *p *in that ordering. We also introduce the uptags of *t*, *Up*(*t*) and the downtags of *t*, *Down*(*t*). *Up*(*t*) is the set of *k *highest ranked probes in *probes*(*t*), i.e., the most upexpressed as compared to control, and *Down*(*t*) is the set of *k *lowest ranked probes in *probes*(*t*), i.e., the most downexpressed as compared to control. The parameter *k *is adjustable and is defined by the user.

### Comparing Rankings

We are interested in finding drugs with similar therapeutic effect by comparing the rankings of probes in gene expression profiles of the drugs. The most straightforward approaches to compare these ranked lists, for example calculating the intersection of *Up*(*q*) and *Up*(*t*), quickly fail when there is any experimental noise. More robust methods are needed to be able to combine and draw conclusions from the large amount of gene expression data that has been created across the world.

#### A Two-sided Approach

A more sophisticated approach to comparing the similarity of two rankings is to compare both the uptags and downtags, and rather than looking simply at the overlap in the sets of tags, take into account the relative ranking of the probe. We will refer to this approach as the *two-sided relative ranking *approach. This type of approach may be able to correctly weight both ends of the ranking and overcome noise in the experimental data.

The CMAP approach [8] is a recently introduced treatment retrieval method that is an example of a two-sided relative ranking approach. Here we explain the CMAP method and ground it in our example domain. The following equations are adapted from Lamb et al. ([8]) and are based on their definition of gene set enrichment [2]. The CMAP method is based on a similarity measure which uses a truncated Kolmogrov-Smirnov (KS) [13] statistic applied to the up and down probes of the treatments. The KS statistic measures the similarity between two distributions; the truncated KS statistic focuses on the tail ends of the distributions. Given a query treatment *q *and target treatment *t*, the KS score is high if a) the probes in *Up*(*q*) tend to also be highly ranked in *t*, b) the probes in *Down*(*q*) tend to have low ranks in *t*, and finally c) the probes in *Up*(*q*) tend to be more highly ranked in *t *than the probes in *Down*(*q*). This is similar to the truncated statistical approach seen in [14] in the whole genome association study in search of genetic markers for continuous traits.

The KS statistic of treatment instance *t*, given a query instance *q*, *KS*(*t, q*), is computed using the uptags and downtags of *q *and the full ranking of all the probes in *t*. *KS*(*t, q*), in turn, is computed from two separate statistics, *KS*_*u*_(*t, q*) and *KS*_*d*_(*t, q*), which are calculated on the uptags and downtags of *q *respectively.

*KS*_*u*_(*t, q*) measures where the uptags of the query are located within the distribution of probes in a treatment instance *t*. It is a number between -1 and 1. If it is close to 1, it tells us that the uptags of *q *are also highly ranked in *t*, or more specifically that the probes that are most upexpressed in the query instance also tend to be upexpressed in the treatment instance.

In order to compute *KS*_*u*_, based on the selected set of probes, *Up*(*q*), we define *Up*_*t*_(*q*) to be the probes in *Up*(*q*) sorted according to their rank in *t*, *r*(*p, probes*(*t*)). Next we define the rank of *p *in this new sorted set of probes:

We introduce shorthand *p*_*t *_= *r*(*p, probes*(*t*)) and *p*_*q *_= *r*(*p, Upt*(*q*))

Now we have the required information to compare the probe distributions between the query and each treatment. Let

and

*KS*_*u *_is computed as follows:(1)

*KS*_*d *_is calculated analogously using *Down*(*q*). To compute *KS*_*d*_, based on the selected set of probes, *Down*(*q*), we define *Down*_*t*_(*q*) to be the probes in *Down*(*q*) sorted according to their rank in *t*, *r*(*p, probes*(*t*)). Next we define the rank of *p *in this new sorted set of probes:

We assign *p*_*q *_= *r*(*p*, *Down*_*t*_(*q*)) and calculate *a*, *b *and *KS*_*d *_as before.

Finally we can calculate the truncated KS statistic using the *KS*_*u *_and *KS*_*d *_as follows:(2)

Referring back to our original description of the properties that we were looking for in the KS statistic we see that when the sign of *KS*_*u *_and *KS*_*d *_are the same, whether both positive or both negative, then the KS score is set to zero. This indicates that there is a significant overlap between the two distributions. No clear separation means that the two distributions are randomly dispersed, and that this ranked list is not statistically similar to the query sequence. In the case where the sign of the two values is different then the final KS score represents the separation between the two distributions. This is done by calculating the difference between *KS*_*u *_and *KS*_*d*_.

The CMAP approach was developed as a query system that directly compares the query to each treatment in the database. It does not take into account any further information about how the treatment instances in the database relate to each other. We refer to this as a *direct *approach.

#### Indirect Two-sided Approach

**Figure 1:**
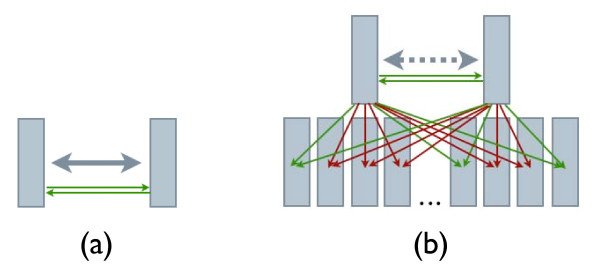
**Direct vs. Indirect Similarity**. (a) The direct similarity method evaluates a given pair of instances without taking into account any other knowledge in the database. (b) The indirect similarity method benefits from the extra knowledge within the database, comparing the similarity of the pair to all the instances in the database and uses this information to determine the similarity between a given pair of instances.

Next, we introduce an *indirect two-sided relative ranking *method which compares the similarity between the query and treatment instance by comparing their corresponding similarity to *all *the instances in the database. Ideally, by combining hundreds, thousands, or even millions of pairwise distances, a more robust similarity measure can be obtained. This is similar in spirit to a vantage point method for computing similarity in metric spaces, where the distance between a pair of points is computed based on their distance to a collection of vantage points [15]. An illustration of the difference between our indirect method and a simple direct similarity method is shown in Figure 1.

Our indirect two-sided relative ranking is calculated by comparing the correlation between how two treatments compare to the rest of the database. There are many correlation measures, including parametric statistics such as Pearson coefficient and nonparametric statistics such as Spearman rank correlation coefficient. Since we do not know ahead of time if the gene expression data is normally distributed, it is safer to use a nonparametric correlation measure. We use the Spearman rank correlation coefficient.

We compute the Spearman rank correlation coefficient by measuring the difference between the KS statistics for the query *q *and target treatment *t*, for all the treatments in the database D. Let *KS*_*D*_(*q*) = {*KS*(*q*, *t*_1_), *KS*(*q*, *t*_2_), ..., *KS*(*q*, *t*_*n*_)} and let *KS*_*D*_(*t*) = {*KS*(*t*, *t*_1_), *KS*(*t*, *t*_2_), ..., *KS*(*t*, *t*_*n*_)}. Then we define the indirect two-sided relative ranking of a query *q *and a treatment *t*, *I*2*R*(*t, q*) to be:

where *Spearman *is the Spearman correlation statistic, which we will formally define next.

Let *r*(*KS*(*q*, *t*_*i*_), *KS*_*D*_(*q*)) be the rank of the score of instance *t*_*i *_(*KS*(*q*, *t*_*i*_)) out of all scores returned (*KS*_*D*_(*q*)) when querying with *q *and analogously let *r*(*KS*(*t, t*_*i*_), *KS*_*D*_(*t*)) be the rank of the score of instance *t*_*i *_out of all scores returned when querying with *t*.

where *n *is the number of instances in the database. This score is calculated using the ranks of all of the pairwise KS scores from *q *and *t *to each other instance *t*_*i*_. This is equivalent to taking the Pearson's correlation over the ranks. In the case where there are tied ranks, the full Pearson's correlation over ranks must be calculated. The indirect similarity score is therefore a calculation of how two instances individually compare to the full database (inclusive of the query and target). If they tend to be similar, or dissimilar, to the same instances, then they are more likely to be similar to each other.

An advantage of this method is that it can build on any individual pairwise similarity available. Here we have taken what we believe to be the current best method, the KS statistic from the CMAP approach, and used this as our source of pairwise similarities. If other direct similarity measures for this domain become available, we can easily incorporate them. Another advantage of this method is that as more treatments are added to the database, additional evidence is available, which can further increase the accuracy of our indirect similarity calculation.

## Discussion

As mentioned at the outset, one of the tasks that we are interested in is finding similar treatments by comparing the gene expression profiles of drugs. Specifically, our goal is to improve the ability to detect similarity in the presence of experimental noise. We focus our evaluation on the case where we have known experimental noise, e.g., when 1) the samples come from different vehicles, 2) they belong to different batches, or 3) they differ in both vehicle and batch (which corresponds to the most labeled experimental noise). Though vehicle and batch are not the only sources of experimental noise, they can easily be evaluated as they are both annotated.

The ideal outcome of such a discovery program is the in vivo validation of a drug predicted by gene expression similarity to be useful for an unknown, alternative indication. To simulate this goal, we propose calculating the recall at rank k of drugs of the same PDR classification. More specifically, we query the database with each drug from a particular indication and we measure the total number of drugs recalled across the top *k *results from all of the individual queries. We measure this recall of drugs which are known to be used for the same indication across vehicles, across batches, and across both vehicles and batches. We focus our analysis on the most populated PDR classifications, where 10 or more drugs from each group have been profiled, which results in 14 different groups. This filtering of groups is done to avoid unrepresentative results caused by a small sample size. For the evaluation we select recall at rank *k *= 10, but we also demonstrate that these results are not greatly affected by variations in *k*.

### Sample Evaluation

Given the group *Histamine Antagonists*, we calculate the recall at rank 10 as follows:

1. For each histamine antagonist, determine the 10 most similar compounds using each method (direct vs indirect).

2. Count the number of compounds of the same class, i.e., Histamine Antagonist, that are screened in a different vehicle and different batch.

3. Improvement of indirect over direct is represented as:

where [indirect] and [direct] are the number of recalled treatments from the top 10 of each method respectively.

4. When [direct] = 0, and [indirect] ≥ 0 then we make note of this improvement as a special case. Reporting percent improvement does not make sense when the baseline is 0, so we do not include these in our overall improvement calculation, but we note them as they are very important special cases where the existing approach returns nothing, while our approach returns useful results.

The results of our example of comparing the two similarity methods for recall at rank 10 across both different vehicles and different batches for the Histamine Antagonists are shown in Table 1. It is important to note that these are the total number of drugs recalled across all of the histamine antagonists used to independently query the database. In this case, the direct method finds two results while indirect finds three, which is an improvement of 50%. This example analysis compares the ability to recall other histamine antagonists across both vehicles and batches.

**Table 1:** Example evaluation comparing the direct and indirect methods.

Number of results returned by direct	2
Number of results returned by indirect	3

Improvement of indirect over direct	50%

Using the evaluation criteria presented above, we compare the ability of the two methods (direct and indirect) to overcome experimental noise. We evaluate how these methods work on two different datasets within the drug discovery domain. The first is a large, proprietary dataset (GEPedia) from Vanda Pharmaceuticals. This dataset contains a large number of drugs that have been profiled. While this dataset is not currently in the public domain we feel that it is worth describing these results as they demonstrate that this method is in fact being employed in a real world drug discovery engine. Realizing that this analysis provides only anecdotal evidence of this method, we therefore also include an evaluation on a similar public dataset from the Broad Institute http://www.broad.mit.edu/cmap which contains 453 profiles. It is important to note that this dataset includes a substantial amount of replicates for many of the compounds.

### Vanda GEPedia Summary Results

We start by comparing the two methods, direct and indirect, using the Vanda GEPedia dataset. Once again, these results are being provided as anecdotal evidence of how this method performs in a real drug discovery engine. The average recall at rank 10 for the 14 PDR groups is presented in Table 2. The indirect method improves over the direct method and is able to recall 71.44% more true positives when searching across different vehicles. The positive predictive value (PPV) increases from 2.0% for the direct method to 2.3% for the indirect method. The PPV is calculated for each group independently; we present the average PPV across all of the groups. The improvement in recall of known similar drugs is increased when searching across batches to 94.93% (Direct PPV of 2.1% vs. Indirect PPV of 2.7%). When attempting to detect similarity across both vehicles and batches, which represents the most experimental noise in our setup, the indirect method has an improvement in recall of 97.03% as compared to the direct method (Direct PPV of 1.3% vs. Indirect PPV of 1.7%). While the PPV appears low for both methods, it is important to realize that the expected discovery rate of a drug engine is inherently low, and that our evaluation method only considers already labeled true positives. Additionally, other steps could be taken to determine which candidates to move forward including for example, obtaining further evidence from literature, desired characteristics for the particular indication (e.g., the ability to cross the blood-brain barrier), or conducting in vitro experiments. The indirect similarity method recalls almost twice the amount of true positives (similar drugs) as the direct method. This level of improvement brings the potential for important scientific discovery and impact of such a system.

**Table 2:** Percent improvement of indirect similarity recall over direct similarity recall in different conditions.

Across Different Vehicles	71.44%
Across Different Batches	94.93%

Across Different Vehicles & Batches	97.03%

As mentioned earlier, the average percentage improvement does not capture the important special case that occurs if one of the methods does not retrieve *any *treatments. These special cases are further examples of the ability of the indirect similarity method to detect similarity when the direct method cannot. These cases are listed below for those found across different vehicles, across different batches, and lastly across both different vehicles and different batches (Table 3).

**Table 3:** Instances found in conditions where the direct method had found none.

PDR Class	Vehicle	Batch	Vehicle + Batch
Antibiotic	12	11	8

Anesthetic	1	1	1

Antihypertensive	1	1	0

Anticonvulsant	0	2	2

We have shown how overall, the indirect method which uses the Spearman rank correlation has a higher recall at rank 10 than the direct KS method.

### Vanda GEPedia In Depth Analysis

Next we study the three largest groups (the groups with the most compounds profiled) in more detail: Antibiotic, Histamine Antagonist, and Analgesic, in order to a) further inspect the differences in the results returned by each of the methods and b) to verify that this is not an artifact of using *k *= 10.

#### Antibiotic

The Antibiotic group has the largest amount of compounds in the database (*n *= 58). This group is in the PDR class *Anti-Infective *and the PDR subclass *Antibiotic*. An antibiotic drug is one that inhibits the growth of micro-organisms. The indirect method is able to recall eight antibiotics when searching across both vehicle and batch, compared to 0 recalled by the direct method. This result is not driven by any single treatment, i.e., each of these 8 recalled treatments is not only unique, but they are also recalled by distinct query treatments.

**Figure 2:**
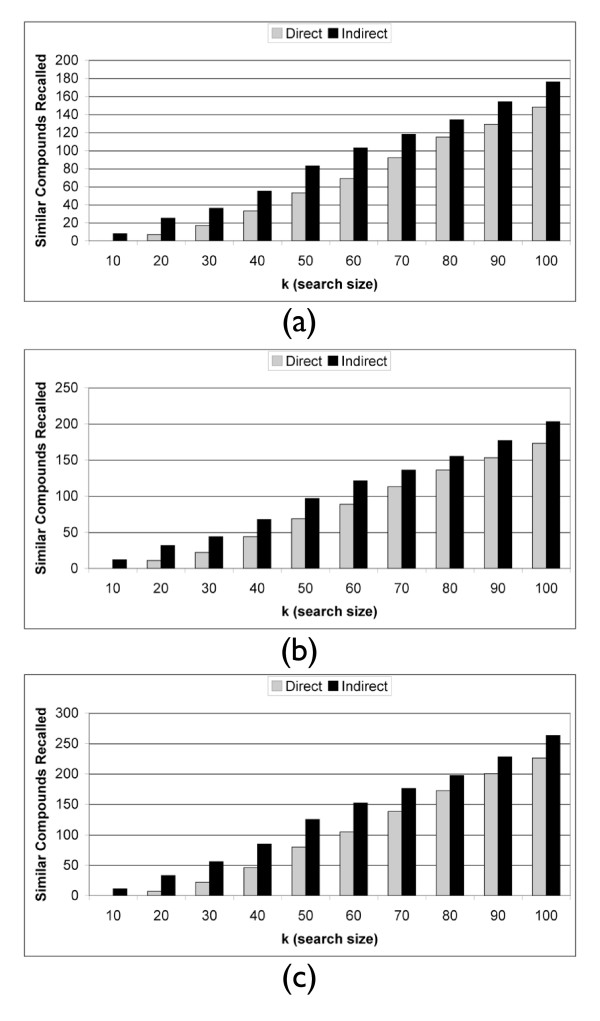
**Antibiotic Recall**. Antibiotic recall at rank *k *across (a) both different vehicles and batches, (b) different vehicles, and (c) different batches. There are *n *= 56 Antibiotic instances in this dataset.

Next, we demonstrate that these results are not biased by our selection of *k *= 10. Figure 2(a) shows the recall of the two methods across both different vehicles and batches with values of *k *ranging from 10 to 100. The indirect method is able to recall more true positives independent of k. We can also evaluate how the methods compare when searching over vehicle or batch separately. Figure 2(b) shows that when searching across different vehicles only, the same trend is seen as in Figure 2(a). Similarly, evaluating the two methods when searching across different batches (Figure 2(c)), a similar trend is seen in which the indirect method outperforms the direct method regardless of *k*.

#### Histamine Antagonist

The Histamine Antagonist group contains the second largest number of compounds profiled (*n *= 24). This group is made up of drugs in the PDR class *Respiratory Agent *and PDR subclass *Histamine Antagonist*. A histamine antagonist inhibits the release or minimizes the action of histamine. There are several subtypes of histamine antagonist based on their binding affinity to the different histamine receptors. The *H*_1 _receptor antagonists, sometimes referred to as antihistamines, are clinically used to treat allergies. The other common subtype, the *H*_2 _receptor antagonist, are commonly used to control the secretion of gastric acid. There are other subtypes, namely *H*_3 _and *H*_4_, however, they are not often used clinically. Once again we do not distinguish between these subtypes for our analysis; we use the PDR classification.

**Figure 3:**
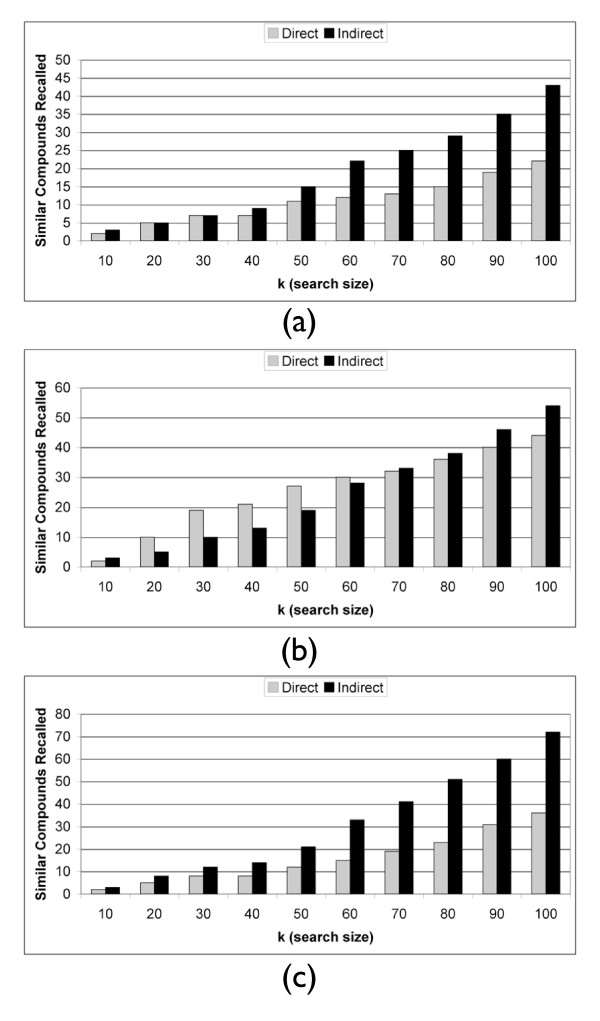
**Histamine Antagonists Recall**. Histamine Antagonist recall at rank *k *across (a) both different vehicles and batches, (b) different vehicles, and (c) different batches. There are *n *= 24 Histamine Antagonist instances in this dataset.

As discussed earlier, for the histamine antagonists, the direct method is able to recall two antihistamines at or below rank 10 while the indirect method is able to recall three. This corresponds to an increase of 50%. More specifically, the indirect method recalls the same two treatments as the direct method in addition to a third novel treatment. Figure 3(a) shows the ability of each method to recall histamine antagonists across both different vehicles and batches. The recall at rank 20 and at rank 30 is the same for the two methods, and then as *k *increases the indirect method improves in its ability to recall histamine antagonists as compared to the direct method. In splitting up the vehicle (Figure 3(b)) and batch (Figure 3(c)) analysis, we see that the direct method is outperforming the indirect method in the across vehicle analysis for smaller *k*, while underperforming against the indirect method in the across batch analysis, which contains more instances.

#### Analgesic

The last group that we individually analyze is the Analgesic group (*n *= 23). This group is defined as drugs belonging to the PDR class *Central Nervous System Agent *and PDR subclass *Analgesic*. An analgesic, more commonly known as a painkiller, acts in various ways on the peripheral and central nervous system in order to reduce pain. Searching across both vehicles and batches the direct method is able to recall one analgesic. The indirect method recalls the same treatment in addition to two other treatments. The indirect method is able to recall three analgesics in total which corresponds to a 200% percent increase in recall.

**Figure 4:**
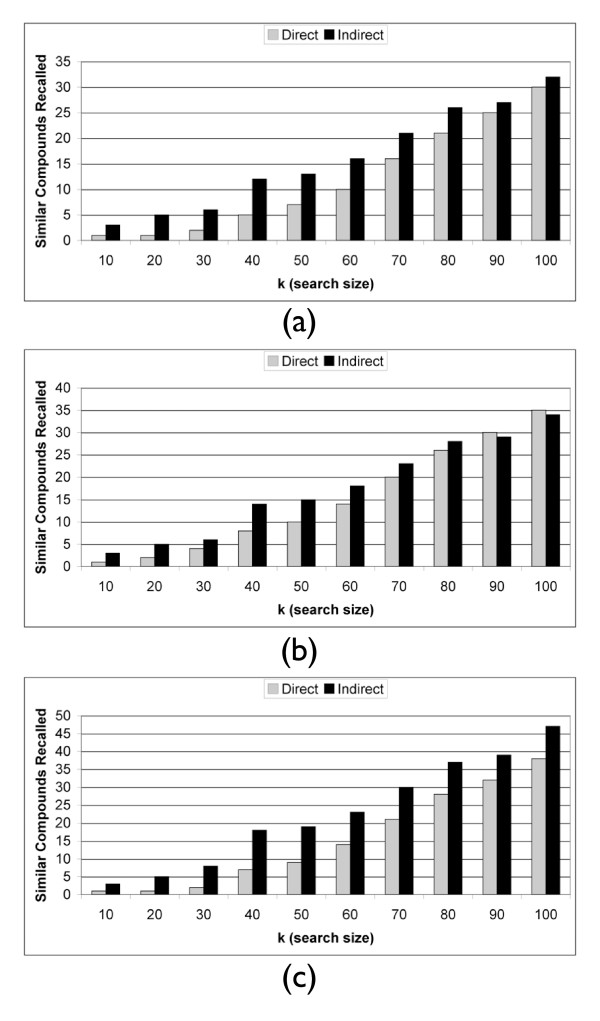
**Analgesic Recall**. Analgesic recall at rank *k *across (a) both different vehicles and batches, (b) different vehicles, and (c) different batches. There are *n *= 23 Analgesic instances in this dataset.

Figure 4(a) shows that the indirect method has a higher recall rate at every level of *k *when searching across both vehicle and batch. The same is true when searching across just a different batch (see Figure 4(c)). We see in Figure 4(b) that the indirect method also does better for low *k *across different vehicles. It is more important for a method to do better for low *k *because in a drug discovery system you will start validation on the most promising hits first. It quickly becomes cost prohibitive to explore a large set of leads.

### Broad Dataset

Next, we replicate our findings using the publicly available gene expression dataset from the Broad Institute. This dataset consists of 453 samples and was released with the Connectivity Map tool. To allow for easier reproducibility, we make use of the annotations provided on the CMAP website as opposed to custom matching to the PDR annotations. In terms of PDR indications we instead use what is described as *Therapeutic Uses *in the ChemBank [16] record linked to each CMAP instance. There is no information provided about the vehicles used for each sample. However, each instance is associated with a batch, and so we will use this information to segment our data. To remain consistent and in order to have more confidence in the results, only groups (Therapeutic Uses) with 10 or more instances are used. The groups, along with the number of instances in each group, are listed in Table 4.

**Table 4:** Description of therapeutic groups within the Broad Dataset.

Therapeutic Use	Number of instances
anti-inflammatory	28
anti-convulsant	21
anti-psychotic (neuroleptic)	19
anti-proliferative	16
tranquilizer	16
anti-neoplastic	15
analgesic	13
immunosuppressive agent	13
cardiovascular agent	10

Similar to what was observed before, the indirect similarity measure recalls more compounds of the same class than using the direct similarity measure alone. The improvement of the indirect method over the direct method is 49.44% on the Broad dataset. The average PPV of the direct method is 6.2% compared to 7.6% for the indirect method. Once again, the indirect method allows for the ability to recall more true positives, and the improvement is substantial.

We now analyze the three largest therapeutic groups from the Broad dataset. Note that for this set of data we explore a smaller size for *k*. Given that this is a smaller database, we want to guarantee that we are only evaluating the top pairs. We begin our analysis with the Anti-Inflammatory group.

#### Anti-Inflammatory

**Figure 5:**
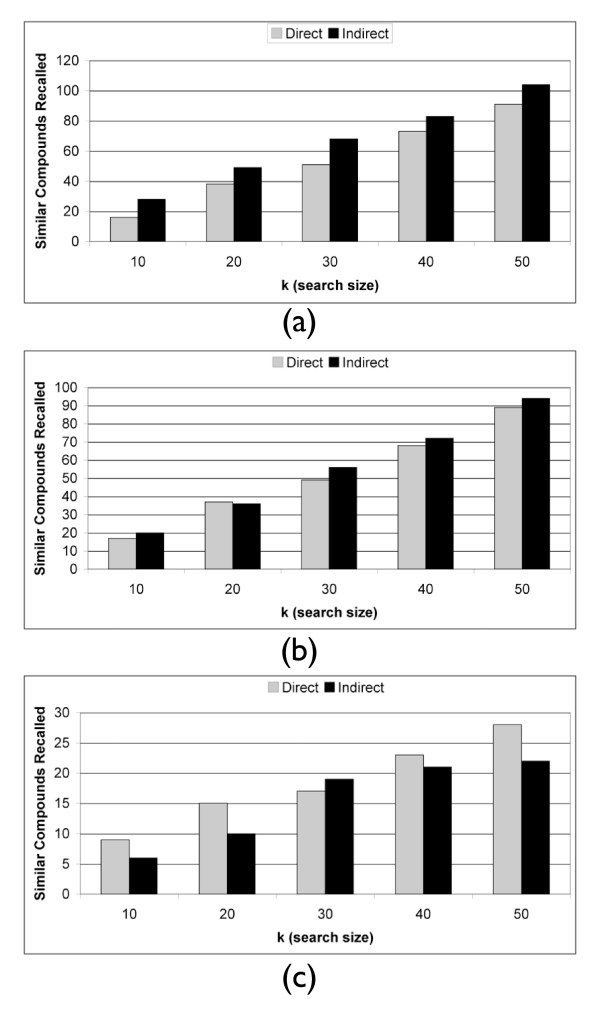
**Broad Validation Recall**. Broad validation results with varying *k *on top 3 groups: (a) Anti-Inflammatory (*n *= 28) (b) Anti-Convulsant (*n *= 21), and (c) Anti-Psychotic (*n *= 19).

To illustrate this improvement, let us look at the group with the most compounds: the anti-inflammatory group. An anti-inflammatory drug is a substance that reduces inflammation. Many analgesics are anti-inflammatory agents, alleviating pain by reducing inflammation. The direct approach is able to recall 16 compounds labeled as anti-inflammatory that have been profiled in a different batch (*k *= 10). The indirect approach, however, is able to recall 28 compounds that also are classified with a therapeutic use of anti-inflammatory. The improvement of the indirect method over the direct method can be seen in Figure 5(a). We see that the indirect method is always better than the direct method, and this improvement is even more pronounced with lower *k *values.

#### Anti-Convulsant

The second group that we will focus on in the Broad validation dataset is the anti-convulsant group. Anti-convulsant drugs are used in the prevention and treatment of epileptic shock. The mechanism by which these drugs work is by suppressing the rapid firing of neurons. At *k *= 10 the direct method has a recall of 17 while the indirect method is able to recall 20 other anti-convulsants (from different batches). Figure 5(b) shows that this trend generally holds for this group, with a slight dip at *k *= 20.

#### Anti-Psychotic

The last group that we will evaluate is the anti-psychotic group, which has the third highest number of instances in the Broad dataset. Anti-psychotic drugs are used to treat psychosis. Neither method in this group is able to recall as many instances as in the previous two groups. The direct method recalls 9 anti-psychotics while the indirect method recalls 6 (*k *= 10). The indirect method is able to gain an advantage at *k *= 30, however, then the methods switch back again (Figure 5(c)). The recall of both methods is much lower for this anti-psychotic group (in the Broad dataset) than previous groups and this is a possible explanation for why we do not see improvement.

### Evaluating the Statistical Significance of Improvement

We have demonstrated that our indirect method results in a large improvement in the recall of similar compounds over the direct method in the face of vehicle and batch effects. Specifically, we have shown an improvement of known true positives at rank 10 across a number of therapeutic groups. We evaluate if the difference in ranks of these true positives is statistically significant. Our indirect method does statistically better than the direct (CMAP) approach in 33% of the groups while remaining as accurate as the direct method on the remaining groups. This analysis is performed for the groups listed in Table 4 as follows. For each set of instances belonging to a particular therapeutic group we use both methods (direct and indirect) to determine the top 100 similar instances per method. We avoid cases where neither method recalls the true positives within the top 100 instances, as differences this far down the result listing is of limited practical interest. However, if one method recalls an instance within the top 100, we include the rank for the other method even if it is outside of the top 100, because we want to give either method credit for these cases. We perform a paired t-test between the two methods for each of the therapeutic groups. The indirect method is statistically better in 3/9 of the therapeutic groups when evaluating the top 100 results and is statistically equivalent (no statistical difference between the methods) in the other 6 groups. In selecting 100 as the threshold we evaluate at several other thresholds as well. The indirect method is never statistically worse than the direct method across all 900 evaluations (9 groups × 10 rank thresholds) and is statistically better in 22 cases, including being statistically better at all thresholds 20-100 for the anti-inflammatory group, which is the biggest group with 28 instances. The results for this analysis at a search threshold of 100 are show in Table 5 and the followup sensitivity analysis is shown in Table 6.

**Table 5:** Statistical Analysis of the Improvement in Rank

Therapeutic Use	N	Mean Improvement	StdDev	tValue	P
analgesic	29	90.43	117.55	4.14	0.0003
anti-inflammatory	160	30.12	115.04	3.31	0.0011
anti-psychotic (neurole)	69	25.57	73.36	2.90	0.0051
immunosuppressive agent	67	7.96	40.42	1.61	0.1120
tranquilizer	68	14.43	73.95	1.61	0.1124
cardiovascular agent	22	10.82	69.37	0.73	0.4726
anti-neoplastic	54	10.33	114.65	0.66	0.5106
anti-convulsant	144	-4.50	92.09	-0.59	0.5585
anti-proliferative	86	-1.86	77.86	-0.22	0.8252

A paired t-test was performed to analyze the difference in the rank of true positives returned by the direct and indirect method. *N *is the number of true positives found within the top 100 results across the whole therapeutic group. Improvement is the average improvement (rank direct - rank indirect).

**Table 6:** Statistical Analysis of the Improvement in Rank

Search Size:	10	20	30	40	50	60	70	80	90
Therapuetic Use									
analgesic	0.136	0.087	0.041	0.020	0.001	2.0E-04	0.004	0.001	0.002
anti-convulsant	0.811	0.529	0.932	0.636	0.918	0.856	0.478	0.481	0.512
anti-inflammatory	0.151	0.040	0.034	0.033	0.002	0.001	0.003	0.002	0.004
anti-neoplastic	0.412	0.842	0.943	0.928	0.577	0.804	0.919	0.909	0.779
anti-proliferative	0.148	0.553	0.778	0.659	0.987	0.881	0.942	0.837	0.837
anti-psychotic (neurole	0.905	0.852	0.686	0.674	0.529	0.129	0.063	0.024	0.006
cardiovascular agent	0.647	0.267	0.496	0.496	0.540	0.795	0.984	0.801	0.687
immunosuppressive agent	0.047	0.664	0.642	0.550	0.525	0.525	0.554	0.323	0.323
tranquilizer	1.000	0.580	0.579	0.921	0.665	0.619	0.413	0.290	0.159

A sensitivity analysis using multiple paired t-tests was performed to analyze the difference in the rank of true positives returned by the direct and indirect method at different search thresholds for rank *k*. Each cell contains the respective probability. All significant (*P *< .05) findings are confirmed to be in favor of the indirect method.

### Evaluation For Classification on Additional Datasets

We have demonstrated how this novel method can work in a large, gene expression based, drug discovery framework which has been our motivating problem and focus. We now analyze our indirect method on three smaller (public) datasets. We evaluate how our indirect method performs in distinguishing cancer types (acute myeloid leukemia versus acute lymphoblastic leukemia) and in predicting drug sensitivity/resistance. Additionally, we demonstrate the ability to use our indirect method to distinguish three very similar and related cell types in a third dataset.

#### Molecular Classification of Cancer

Golub et al. [3] evaluated the use of gene expression signatures to classify acute leukemias. They created a database of expression profiles of both acute myeloid leukemia (AML) and acute lymphoblastic leukemia (ALL) samples and demonstrated how gene signatures can help to classify these subtypes of acute leukemia. This is an important task, as the appropriate treatment for an individual depends on understanding the tumor type. Maximizing efficacy and minimizing adverse events and toxicity is the goal, and this is best achieved by prescribing chemotherapies that target the correct pathogenetically distinct tumor types.

This dataset consists of 52 samples (24 ALL and 28 AML). Analogous to searching for similar drugs we can search for samples of the same cancer class, e.g., searching with an ALL sample should yield other ALL samples. In order to perform the classification we use the majority vote of the top *k *results. In this case, the majority vote of the top 11 results (as opposed to 10 to avoid ties) recalled by a given sample is used to classify the sample. In this example, the direct method does extremely well, accurately classifying every sample correctly. The indirect method also correctly classifies every sample correctly. However, this is a high level comparison and we can understand and evaluate the results in more detail by looking at the individual rankings upon which the voting relies.

**Figure 6:**
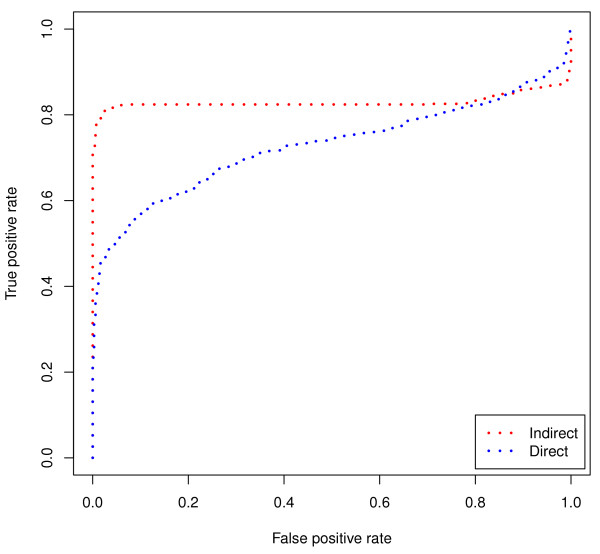
**AML versus ALL ROC**. ROC curve showing the difference in sensitivity and specificity between the direct and indirect method for the task of predicting subtypes of cancer (AML versus ALL). The indirect method (shown in blue) performs better than the direct method (shown in red). The difference is statistically significant (p = 0.0001 for the paired t-test on the underlying ranks).

We describe the average rank (of samples of the same class) across the two methods. The average rank for ALL by the indirect method is 12.2 compared to 14.5 for the direct method. The AML class also demonstrates an improvement where the average rank for the indirect method is 20.5 versus 21.6 for the direct method. While this improvement is consistent across both groups, the small number of groups in this dataset does not readily allow us to evaluate the statistical significance. For this we instead evaluate the underlying ranks for each sample. The full results are listed in Table 7 and Figure 6 shows the corresponding ROC curve. The AUC for the 0.829 for the indirect method and 0.727 for the direct method. This difference is statistically significant using the method described by DeLong et al [17] (p = 1.13e-32). Note that *k *is set to 250 to compensate for there being roughly half of the probes as used in the previous datasets (12, 564). The average rank improvement of recalling similar samples is 1.7 when using the indirect method as compared to the direct method.

**Table 7:** AML vs ALL Average Rank

Sample	Class	Indirect	Direct
ALL 1	ALL	12.0	13.3
ALL 2	ALL	12.0	14.1
ALL 3	ALL	12.1	19.2
ALL 4	ALL	12.0	14.3
ALL 5	ALL	12.0	15.4
ALL 6	ALL	12.0	12.7
ALL 7	ALL	12.0	12.1
ALL 8	ALL	12.0	16.0
ALL 9	ALL	12.0	12.3
ALL 10	ALL	12.0	12.3
ALL 11	ALL	12.0	14.1
ALL 12	ALL	12.0	12.0
ALL 13	ALL	12.2	16.4
ALL 14	ALL	12.0	13.0
ALL 15	ALL	12.0	13.0
ALL 16	ALL	12.0	16.9
ALL 17	ALL	12.1	14.8
ALL 18	ALL	12.0	13.1
ALL 19	ALL	12.0	14.1
ALL 20	ALL	17.0	21.3
ALL 21	ALL	12.0	13.5
ALL 22	ALL	12.0	13.3
ALL 23	ALL	12.0	14.1
ALL 24	ALL	12.0	17.4
AML 1	AML	18.5	20.7
AML 2	AML	18.3	19.5
AML 3	AML	18.3	19.9
AML 4	AML	18.3	19.2
AML 5	AML	18.6	21.7
AML 6	AML	18.4	20.3
AML 7	AML	18.3	19.3
AML 8	AML	18.4	20.7
AML 9	AML	18.3	19.6
AML 10	AML	18.4	21.4
AML 11	AML	20.4	25.9
AML 12	AML	18.3	19.7
AML 13	AML	18.4	20.7
AML 14	AML	18.2	19.1
AML 15	AML	18.5	20.8
AML 16	AML	18.2	19.4
AML 17	AML	18.5	20.7
AML 18	AML	18.4	20.3
AML 19	AML	18.2	19.9
AML 20	AML	18.3	19.9
AML 21	AML	18.3	19.8
AML 22	AML	18.4	20.3
AML 23	AML	18.3	19.6
AML 24	AML	34.3	28.1
AML 25	AML	34.3	28.5
AML 26	AML	33.9	28.3
AML 27	AML	34.3	30.4
AML 28	AML	14.0	22.4

The average rank of samples of the same class is listed for each sample. (ALL = acute lymphoblastic leukemia; AML = acute myeloid leukemia)

#### Predicting Drug Sensitivity/Resistance

**Figure 7:**
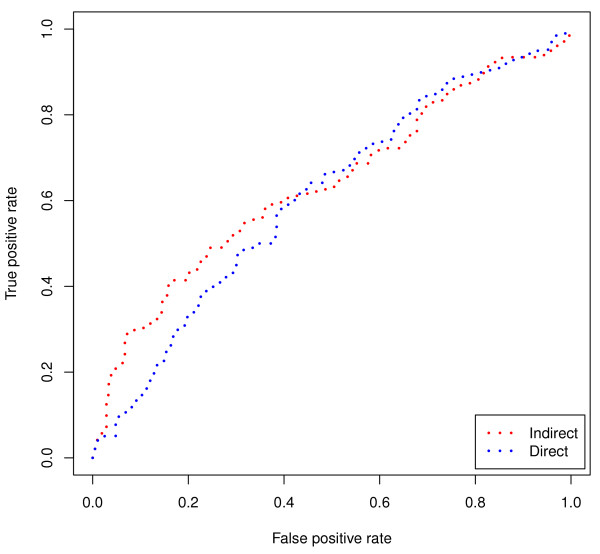
**Glucocorticoid Sensitivity/Resistance ROC**. ROC curve showing the difference in sensitivity and specificity between the direct and indirect method for the task of predicting sensitivity or resistance to glucocorticoids. The indirect method (shown in blue) improves upon the direct method (shown in red). The difference is statistically significant (p = 0.0156 for the paired t-test on the underlying ranks).

The next dataset (from Wei et al. [18]) that we evaluate consists of ALL expression profiles of individuals that are known to be sensitive or resistant to glucocorticoid treatment, specifically in regards to childhood ALL. This is an important task because a poor prognosis is linked to resistance to glucocorticoid-induced apoptosis of primary lymphoblastic leukemia cells in vitro [19-22]. There are 13 glucocorticoid sensitive samples and 16 that are glucocorticoid resistant (total n = 29). As before, we use a paired t-test comparing the average rank of recalling samples from the same class (i.e., sensitive or resistant). The indirect method improves upon the direct method by 0.45 on average across all samples. There are 22, 283 probes used in this dataset and *k *is set to 500. The full results are listed in Table 8 and the ROC curve is shown in Figure 7. The AUC is 0.635 for the indirect method and 0.608 for the direct method (p = 1.90e-06).

**Table 8:** Glucocorticoid Sensitivity/Resistance

Sample	Class	Indirect	Direct
DT2004021428-738	S	10.1	12.6
DT2004021429-976	S	9.8	10.5
DT2004021430-1047	S	10.0	10.6
DT2004021431-1219	S	13.6	12.8
DT2004021432-1241	S	13.3	12.1
DT2004021433-1299	S	9.0	9.6
DT2004021434-1307	S	7.9	8.7
DT2004021435-1477	S	7.3	7.8
DT2004021436-1533	S	12.5	12.2
DT2004021437-1553	S	12.4	13.1
DT2004021438-1657	S	14.7	14.0
DT2004021439-1684	S	10.2	9.5
DT2004021440-1696	S	12.1	13.2
DT2004021441-329	R	11.0	12.1
DT2004021442-557	R	9.8	10.7
DT2004021443-685	R	16.8	15.7
DT2004021444-789	R	12.1	12.7
DT2004021446-865	R	20.3	19.2
DT2004021448-1466	R	13.3	13.9
DT2004021449-1652	R	13.3	14.8
DT2004021451-1755	R	11.9	12.7
DT2004021452-2078	R	11.9	13.3
DT2004021453-2200	R	16.5	15.7
DT2004021454-2209	R	14.1	14.5
DT2004021455-vu8978	R	12.0	13.3
DT2004021456-vu9023	R	10.2	11.8
DT2004021457-vu9573	R	10.5	10.7
DT2004021458-vu9728	R	12.0	13.3
DT2004021459-vu9951	R	13.9	14.6

The average rank of samples of the same class is listed for each sample. (S = Glucocorticoid Sensitive; R = Glucocorticoid Resistant)

#### Classifying (Related) Cell Types

The final dataset that we use to evaluate our indirect method is from Lu et al. [23]. It consists of megakaryocyte-erythrocyte progenitors (MEP) as well as the two cell types that MEPs can differentiate into, namely megakaryocytes and erythrocytes. Megakaryocytes are bone marrow cells that are responsible for the production of platelets while erythrocytes are red blood cells. We refer to this dataset as the MEP dataset. The original focus of Lu et al. [23] was to better understand the differentiation process and was not evaluating the classification of these three cell types. There are 320 probes, and we set *k *= 10 in order to maintain roughly the same ratio as before.

**Figure 8:**
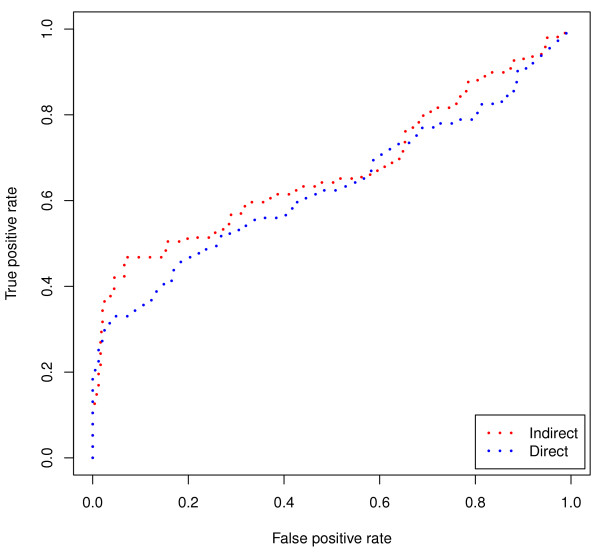
**MEPs (megakaryocyte-erythrocyte progenitors) ROC**. ROC curve showing the difference in sensitivity and specificity between the direct and indirect method for the task of classifying cell types (megakaryocyte, erythrocyte, and their corresponding progenitors, i.e., megakaryocyte-erythrocyte progenitors). The indirect method (shown in blue) outperforms the direct method (shown in red). The difference is statistically significant (p = 0.0035 for the paired t-test on the underlying ranks).

This is another example of how the indirect method can improve over the direct method even with a small dataset. There are only 3 classes and 27 total samples of which 9 are erythrocytes, 10 are megakaryocytes and 8 are MEPs. Analyzing the results in the same fashion as before we find that the indirect method statistically improves upon the direct method once again with an average improvement of 1.1. The ROC curve is shown in Figure 8 with the full listing of results in Table 9. The AUC for the indirect method is 0.670 compared with 0.633 for the direct method (p = 4.13e-13).

**Table 9:** MEPs (megakaryocyte-erythrocyte progenitors)

Sample	Class	Indirect	Direct
ERY1-1	ERY	10.3	13.1
ERY1-2	ERY	9.5	8.8
ERY1-3	ERY	14.0	16.4
ERY1-4	ERY	10.3	14.9
ERY2-1	ERY	16.4	16.4
ERY2-2	ERY	15.8	18.1
ERY2-3	ERY	12.8	13.6
ERY3-1	ERY	18.6	18.4
ERY3-2	ERY	18.6	17.5
MEGA1-1	MEGA	7.3	7.3
MEGA1-2	MEGA	6.9	5.8
MEGA1-3	MEGA	13.7	11.3
MEGA1-4	MEGA	12.1	14.9
MEGA2-1	MEGA	8.2	7.7
MEGA2-2	MEGA	7.0	8.6
MEGA2-3	MEGA	7.3	7.1
MEGA2-4	MEGA	7.4	7.1
MEGA2-5	MEGA	8.7	8.7
MEGA2-6	MEGA	7.4	7.6
MEP-1	MEP	6.1	9.1
MEP-2	MEP	6.0	7.0
MEP-3	MEP	6.3	8.7
MEP-4	MEP	6.1	7.0
MEP-5	MEP	6.1	8.4
MEP-6	MEP	6.3	8.7
MEP-7	MEP	4.7	8.1
MEP-8	MEP	6.1	8.1

The average rank of samples of the same class is listed for each sample. (ERY = erythrocyte; MEGA = megakaryocyte; MEP = megakaryocyte-erythrocyte progenitors)

### Computational Complexity

We have presented a comparison of our novel indirect similarity method to the normal direct similarity method in terms of increased true positives recalled at a given threshold. We have thus far ignored the practical challenges that may occur in implementing either of these methods in a production system. The transition to using an indirect method has implications on both the time needed for calculating the similarity, as well as the space needed to store the information required by the system to work efficiently.

One important thing to note is that we are evaluating the difference between the indirect and direct similarity methods in the context of a knowledge discovery framework. In this situation, the system already performs all pairwise similarity comparisons to detect novel insights. This is quite different than the initial goal of the CMAP method, which was first developed as a real-time, query based tool for the web. The actual running time for the direct analysis was ~24 hours to calculate the KS scores versus ~3.6 hours for the indirect similarity calculation. A fair amount of effort was spent optimizing the indirect calculation and the indirect calculation additionally benefited from being run on a highly parallel SAS server optimized for such statistical calculations. We feel that it is more important to evaluate the theoretical computational complexity which follows below. Additionally, this theoretical analysis applies to any new direct method.

#### Time Complexity

We assume that all the data has been preprocessed and is stored as a rank-ordered list, reflecting the difference as compared to control. For the purpose of this analysis, we are interested in the relative complexity of our indirect method as compared to the complexity of the underlying direct method. As our indirect method can use any underlying direct method as its base, we focus on the relative complexity for a general comparison. Let us assume that the computational cost of one individual pairwise comparison using a given direct method is *c*_1_. The complexity of performing all pairwise comparisons using the direct method is shown in Eq. (3).(3)

The implementation of the indirect method similarity takes advantage of reusing this full matrix of direct comparisons and does not naively recalculate any direct similarities. Once again, assuming a small constant, *c*_2_, to calculate the Spearman correlation for a given pair, the time complexity of our indirect similarity method is given in Eq. (4). It should be noted that in our current experimental setup *c*_2 _<*c*_1_, as the computational complexity of the *KS *statistic far outweighs the complexity of the Spearman correlation.(4)

We have managed to keep the time complexity of our indirect similarity method in the same order of magnitude as what is required by the underlying direct similarity method. We next determine the impact that we have on the space complexity of moving to an indirect approach.

#### Space Complexity

The data that needs to be stored is that of the individual ranked lists representing the treatments as compared to their respective control. For our given application, this works out to be *m*·*n*, where *m *is the size of each ranked list (in our case 22, 283) and *n *is the number of treatment instances. We ignore the negligible space requirement needed for one individual direct comparison since this intermediary is not retained. The space complexity is then once again *O*(*n*^2^) and the direct similarities are stored as a *n *× *n *matrix. Analogously, the indirect similarities are also maintained in an *n *× *n *matrix requiring *O*(*n*^2^) space as well. In the current implementation, both of these space requirements are overshadowed by the large space requirements of the initial dataset itself.

## Conclusions

We have proposed a method for similarity search in gene expression data and an evaluation method based on recall at rank k. We have focused on the ability to detect similarity despite known experimental biases, e.g., different vehicles, different batches, or both different vehicles and batches. The improvements in recall are not expected to only help in overcoming such explained experimental effects, but they are in fact representative of the larger set of unknown environmental effects. It can thus be assumed that the indirect similarity measure will be able to overcome unknown changes in gene expression experiments, and is therefore well suited for comparing gene expression data from vastly different data sources.

Furthermore, we have shown that in a large, proprietary dataset, this indirect method is able to overcome experimental noise better and is able to recall a larger number of drugs that are similar to the query drug. More specifically, the indirect method was able to increase the amount of known similar drugs recalled by 97.03% over the direct method in a real world drug discovery program. These results have been validated on a public dataset (Broad), for which the improvement in recall was 49.44%. The difference in improvement is representative of the fact that the Broad dataset is both smaller and less complex, i.e., contains more replicates. The benefit of the indirect method comes from the information in the rest of the database, and therefore the improvement is expected to increase as the size and complexity of the database grows. While the improvement relies on this added complexity in the database we have shown that the indirect method still works on smaller datasets. Specifically, we have demonstrated that the indirect method improves the rank of recalling samples from the same class in three smaller, public datasets and that this improvement is statistically significant. These additional datasets also demonstrate the diverse type of expression data that can benefit from the indirect similarity method.

The indirect method increases the number of true positives recalled at a particular threshold and this ability to recall 50-100% more compounds that are similar (and therefore less false positives), gives researchers a huge advantage in their analysis. In one scenario, using the indirect method may decrease the amount of candidates that might have to be followed up in an experiment or drug discovery system, thereby saving time and effort by not chasing false positives. This in turn allows more confidence in the results generated by such a system. More importantly, it also brings the community an additional step closer to being able to pull together and learn from the large amount of data that exists in the public domain, which continues to grow every day.

However, it is important to acknowledge that there are many potentially avenues for improvement and further research for this problem. One of the advantages of the indirect approach is that it can build upon any direct similarity method. If better direct methods are developed in the future, then the indirect method can be adapted to use such methods. The work presented in this paper has dealt with the task of adapting to and overcoming experimental bias in gene expression data, specifically for the task of comparing two samples directly. Other potential paths of future research could include more complex comparisons, e.g., analyzing groups of compounds together, as well as a more thorough evaluation of selecting the optimal size of *k *for a given dataset.

## Methods

### Vanda GEPedia Dataset Gene Expression Methods

The Vanda GEPedia Dataset was constructed from the ARPE-19/HPV-16 and H4 cell lines which were obtained from ATCC (Manassas, Virginia), and propagated in culture medium according to supplier's specification. Compounds were purchased from Sigma (St. Louis, MO), with the exception of drugs developed by Vanda Pharmaceuticals. Cells were aliquoted to 96-well cell culture plate (~2 × 105 cells/well) and incubated for 24 hrs prior to providing fresh media with drug at a 10 uM.final concentration, or the drug vehicle (water, DMSO, EtOH, or MEtOH). Cells were harvested 24 hrs later and RNA was extracted using RNeasy 96 total RNA protocol (Qiagen) as indicated by the manufacturer. Gene expression profiles were generated with U133A2.0 microarrays following the manufacturer's protocol (Affymetrix, Santa Clara, CA).

### Significance and Specificity

A permutation test can be used to determine the significance of a particular distribution of a set of instances in an ordered list of all instances as was proposed by Lamb et al. [8]. Such a permutation test works as follows. The indirect similarity score is computed for the set of *t *instances of interest, whether it be instances that are replicates or belonging to the same group, in the ordered list of all *n *instances, giving an average indirect score *I*2*R*_0_. Then, for each of *r *trials, a random *t *instances are selected from the full set of *n *instances and the average indirect score for this set is calculated and denoted *I*2*R*_*r*_. The number of times, *s*, that *I*2*R*_*r *_> *I*2*R*_0 _is counted and the frequency of such an event (*s/r*) serves as a two-sided p-value.

Furthermore, the specificity of any results can also be calculated in the same way as was done for the CMAP approach. An outside set of signatures can be used, e.g., from the MSigDB as is the case with the CMAP, to evaluate the uniqueness of the connections that are detected.

## Authors' contributions

LL and LG developed the method and wrote the paper. LL implemented the method and performed the evaluations. Both authors read and approved the final document.
